# Developing Edge AI Computer Vision for Smart Poultry Farms Using Deep Learning and HPC

**DOI:** 10.3390/s23063002

**Published:** 2023-03-10

**Authors:** Stevan Cakic, Tomo Popovic, Srdjan Krco, Daliborka Nedic, Dejan Babic, Ivan Jovovic

**Affiliations:** 1 Faculty for Information Systems and Technologies, University of Donja Gorica, Oktoih 1, 81000 Podgorica, Montenegro; 2 DigitalSmart, Bul. Dz. Vasingtona bb, 81000 Podgorica, Montenegro; 3 DunavNET, Bul. Oslobodjenja 133/2, 21000 Novi Sad, Serbia

**Keywords:** computer vision, convolutional neural networks, deep learning, digital farm management, edge AI, high-performance computing, machine learning, smart farms

## Abstract

This research describes the use of high-performance computing (HPC) and deep learning to create prediction models that could be deployed on edge AI devices equipped with camera and installed in poultry farms. The main idea is to leverage an existing IoT farming platform and use HPC offline to run deep learning to train the models for object detection and object segmentation, where the objects are chickens in images taken on farm. The models can be ported from HPC to edge AI devices to create a new type of computer vision kit to enhance the existing digital poultry farm platform. Such new sensors enable implementing functions such as counting chickens, detection of dead chickens, and even assessing their weight or detecting uneven growth. These functions combined with the monitoring of environmental parameters, could enable early disease detection and improve the decision-making process. The experiment focused on Faster R-CNN architectures and AutoML was used to identify the most suitable architecture for chicken detection and segmentation for the given dataset. For the selected architectures, further hyperparameter optimization was carried out and we achieved the accuracy of AP = 85%, AP50 = 98%, and AP75 = 96% for object detection and AP = 90%, AP50 = 98%, and AP75 = 96% for instance segmentation. These models were installed on edge AI devices and evaluated in the online mode on actual poultry farms. Initial results are promising, but further development of the dataset and improvements in prediction models is needed.

## 1. Introduction

According to the United Nations Food and Agriculture Organization (FAO), the demand for food is expected to grow by 60% between 2010 and 2050, whereas the demand for animal protein will grow by around 1.7% per year, contributing to the growth of the poultry feed market over the upcoming years [1]. The poultry industry production chain should be continuously optimized and streamlined to meet demand, while simultaneously limiting effects on the environment and improving the well-being of the animals during their short lifespan [2]. Poultry farms are faced with continuous and unavoidable challenges, from controlling all the basics of life (food, water, light, air, sanitation, cleaning) to disease outbreaks and disposal of dead animals. Most of these tasks have had to be done manually, or with the help of modern machinery such as decakers, washers, and heaters. However, some tasks, such as predicting and stopping disease outbreaks before they affect large portions of a flock, ensuring animal well-being, and even remotely optimizing the environmental conditions on farms have remained out of reach until recently. Thousands of chickens can be raised in barns simultaneously and the use of digital tools, such as farm management systems supported by the data collected using Internet of Things (IoT) sensors, opens new possibilities to make the life of farmers easier and to provide them with more in-depth information about their animals. IoT-based smart agriculture solutions typically include monitoring of the environmental conditions, which are very important for raising chickens. Measuring air temperature, air humidity, CO_2_, and ammonia levels is required as a basis for a successful production. Additionally, having insight into chicken body temperature, behavior, and vocalization provides the possibility to undertake adequate measures on time. High-performance computing (HPC) plays a key role in supporting the development of integrated applications across the edge-cloud supercomputer layers in addressing critical scientific, engineering, and societal problems [3]. Combining the evolution of IoT and artificial intelligence (AI) technologies with on-farm video or picture recording has made it possible to create more intelligent tools that can reduce losses, and cut down on manual labor, while increasing feed-to-food ratio and reducing mortality rates, among many other benefits.

More specifically, for poultry farms, in order to improve early disease detection and prevent outbreaks and losses, AI-based prediction models can support functions such as the counting of chickens, assessing the growth and homogeneity during the growing cycle, and the timely detection of dead animals. This research work focuses on the use of HPC to create prediction models for chicken detection and chicken segmentation from images taken on the farm. These models would then be used on edge devices as a part of the IoT sensors installed on the farm. Therefore, the main focus of the research is developing edge AI computer vision to enhance smart poultry solutions using HPC and deep learning.

**Figure 1:**
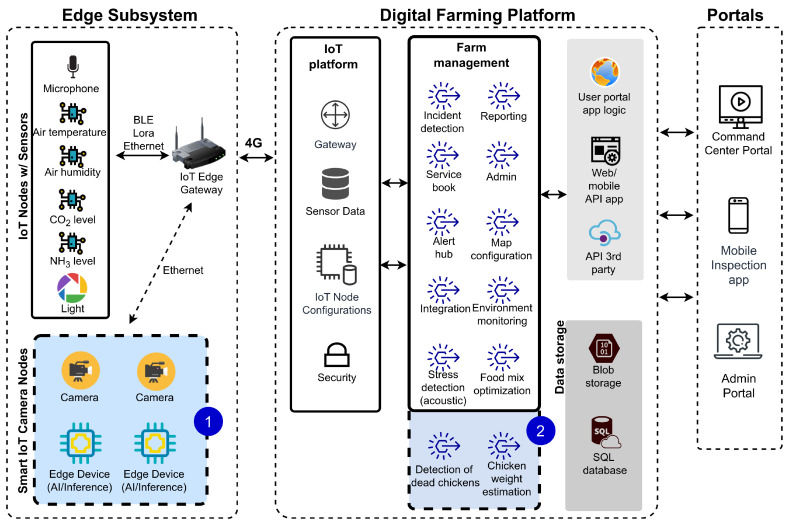
A high-level system architecture of the IoT-based platform for smart poultry farms.

The context of the project is best illustrated with a comprehensive system architecture of the proposed IoT-based digital platform for poultry farms shown in Figure 1. The overall architecture is organized in three distinct layers: (a) the edge subsystem, with sensors and IoT nodes with on-board processing, which is responsible for measurement acquisition and edge computing; (b) the digital farming platform, a cloud-based platform responsible for sensor data collection and aggregation ensuring secure access to data and IoT device management, as well as execution of the business logic, permanent storage of data, and decision support functions; and the (c) portals layer which provides user interfaces in different forms, typically a web application for standard users, a mobile app for farmers while on the move, and a data visualization for advanced users and custom reporting.

In this given context, our research focuses on the use of HPC and AI to develop new functions in the form of smart sensors based on IoT cameras and edge AI devices located in the edge subsystem layer, the section marked with number 1. These smart IoT camera nodes use the prediction models on edge devices and they would send the outputs to the core business platform. New functions of the system that can be derived from new sensor readings can find their place in the digital farming platform layer as a part of the farm management component, labeled as the segment marked with number 2. For example, these functions can include detection of dead chickens and estimation of weight, and together with other sensor data and functions available in the existing platform, can be used to develop solutions for early disease detection and possibly preventing disease spreading.

This paper is an extension of the paper “Developing Object Detection Models for Camera Applications in Smart Poultry Farms” [4] by the same research team. The research is expanded by including both object detection and instance segmentation, and automated machine learning (AutoML) to perform grid search to determine hyperparameters to fine-tune the model training process. This improves the accuracy and helps in selecting an optimal neural network for their use on edge platforms. The research is conducted in the context of the FF4EuroHPC project, a European initiative that helps facilitating access to all HPC-related technologies for SMEs aiming at increasing the innovation potential of European industry [5].

In this paper, we discuss the development of computer vision for smart poultry farm solutions using HPC and deep learning models. The paper is organized as follows: introduction section, related work, materials and methods, results and discussion, and conclusion. The materials and methods section describes the conceptual approach, datasets, selection of software and hardware tools, and experiment setup. The results and discussion section shows the results of the experiment for both object detection and segmentation and gives insight into the system integration and initial field evaluation. The conclusions summarize the main contributions, key results, and benefits, as well as future steps.

## 2. Related Work

Deep learning algorithms are finding their place in image processing and computer vision applications. In recent years, the most mentioned neural network architecture for computer vision is convolutional neural networks (CNNs). However, there are new trends that increasingly highlight the transformer as state-of-the-art architecture, i.e., the visual transformer [6]. For analyzing and detecting objects in images there are two approaches that are often encountered. The first one is the You Only Look Once (YOLO) algorithm [7], and the second is the faster R-CNN algorithm [8]. A general observation is that YOLO is commonly used for real-time detection of objects, when the prediction model takes a very short time period to execute [7]. On the other hand, faster R-CNN is used in situations when precision is more important than the speed of the model, and when detecting smaller objects is important [8]. Another algorithm with the same purpose that offers a balance between prediction speed and model precision quality is single shot multibox detector [9]. The latest versions of the YOLO algorithm promise great speed and improved accuracy when compared to other algorithms. as shown in the comparative analysis given in [10]. As for object segmentation, the mask R-CNN neural network stands out as one of the most optimal [11]. In this research, different variants of faster R-CNN neural networks for object detection and Mask-RCNN for segmentation are analyzed. In [12], the authors provide a detailed analysis of different approaches to solving the segmentation problem. In [13], the authors deal with the implementation of the neural network LC-DenseFCN, focusing on the use of this neural network is to count chickens in an image. The network developed in this paper is based on the combination of two networks, LC-FCN and LC-ResFCN [14]. The chicken detection accuracy achieved by LC-DenseFCN is around 93.84% while the prediction speed is 9.27 frames per second (FPS). A comparison with YOLO [7], EfficientDet [15], and mask R-CNN [11] was provided and for the specific task of counting chickens in an image, and LC-DenseFCN proved to be the fastest and most accurate network. Authors in the [16] deal with the detection and counting of chickens in a cage-free environment. As the authors stated, they implemented the YOLOv5x-hens model based on the YOLOv5x model and reported that the accuracy of the YOLOv5x-hens model in recognizing chickens is about 95% in different environments (age, light intensity, observational angle). As a critical problem, the authors pointed out that the problem of this model is when it is used to detect younger chickens that are about one week old. This is especially problematic when they mix with other objects such as a feeder, but also when they are in a dense flock. In [17,18], the authors compare two key approaches that are used today for object detection, YOLO and Faster R-CNN algorithms. They reported that the advantage of the YOLO approach is the speed of prediction, and in newer versions, the accuracy of the YOLO algorithm may surpass the Faster-RCNN algorithm. A key benefit of the faster R-CNN approach is the more successful recognition of smaller objects. Authors in [18] stated that through YOLOv3, developers tried to overcome the problem of poor detection of smaller objects, but with some compromises, which led to worse recognition accuracy of larger objects when compared to the Faster R-CNN model. The authors in [18] decided to use Faster R-CNN because they had cameras placed at a higher height, thus they needed to detect smaller objects. In addition, the FPS parameter was not so important to them, which is one of the critical advantages of the YOLO algorithm. The paper [19] also describes how the faster R-CNN network can be used to detect chickens and monitor their movement on the farm. They state that they managed to get their method with average precision (AP) 93% accuracy in detecting chickens. The paper [20] uses the YOLOv5 algorithm to detect and track chickens on a farm, and they developed the ChickTrack model, which was tested in different environments. The authors stated that practical uses of the model can be challenging, and after the model testing was done in different environments, mean average precision (mAP) can have a lower value. The authors reported that the value for mAP is around 50%. Object detection and counting are also used in other areas of agriculture for different animals. The authors in [21] deal with counting piglets, and their key idea was to extend the counting CNN model [22]. They showed that they managed to reduce the mean average error (MAE) by a significant value compared to the counting CNN. The work [23] also deals with counting, but in this case, livestock is in the open. They show that mask R-CNN proved to be the best model compared to faster R-CNN, SSD, and YOLOv3. Another interesting paper is [24], which deals with the detection of dead chickens based on a model based on the YOLOv4 algorithm. It stated that the accuracy of this model is slightly above 97%. The paper [25] deals with the assessment of gender ratio in which they state that the accuracy of their model is almost 97%. In the research [26] authors deal with instance segmentation tasks for a goose. They stated that AP50 for both detection and segmentation is 96%. Finally, hyperparameter optimization using AutoML was discussed in [27].

In this paper, we report on the research that focuses on prediction models for processing images collected in poultry farms using faster R-CNN for chicken detection and Mask R-CNN for chicken segmentation. The development of models was done using HPC systems and we specifically dealt with finding the optimal hyperparameters for the models using AutoML.

## 3. Materials and Methods

**Figure 2:**
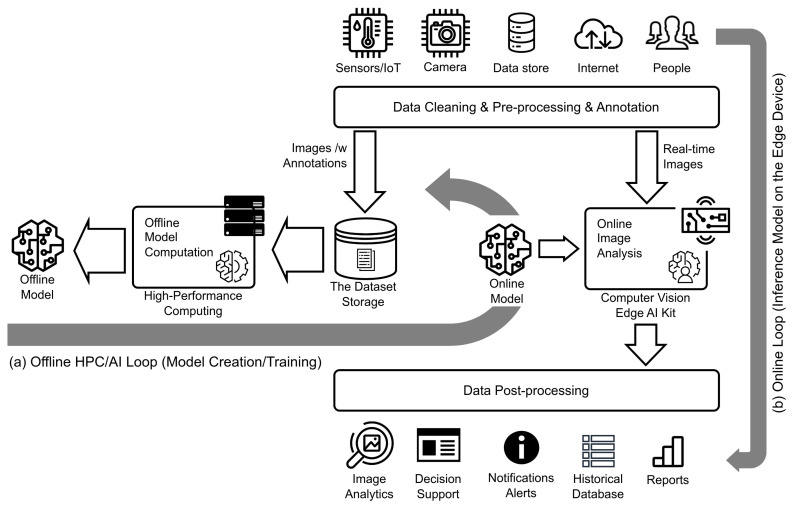
Conceptual approach for the development of computer vision for smart agriculture using HPC and deep learning: (**a**) offline HPC/AI loop: the dataset is used to train and refine prediction models using HPC resources, (**b**) online loop: images captured in the field in real-time processed by trained models ported to the edge AI device.

Figure 2 depicts the conceptual approach used in our study in order to utilize HPC/AI to develop prediction models for smart IoT solutions for farms. This would be a typical computing continuum use case relying on the combination of IoT, edge AI, cloud, and HPC [3]. The setup assumes a presence of an offline loop in which the datasets are used to create, train, and fine tune prediction models, and an online loop responsible for processing images captured in the field in “real-time”, e.g., in a matter of seconds, using trained models on edge AI devices. As shown, input data can come from IoT sensors and cameras, humans and other conventional data sources. In our study, as input data, we used images from cameras installed on poultry farms and input from domain experts needed to create and annotate the datasets. The annotated images were used in the offline loop for model computations on HPC, while the input data for the online loop were only the images coming from the camera sensors installed in farms. The outputs from the prediction models are sent to the digital farming system where they can be used for further analysis, decision support, alerts, and creation of reports.

### 3.1. Datasets

With deep learning algorithms, data is one of the key parameters influencing the quality of the developed model. The key activities to be carried out in this step are the collection, labeling, and processing of data so that it can be used to train deep learning models. For this experiment, the dataset is based on images of chickens extracted from the video material that was recorded in a couple of farms in Serbia and Montenegro. All of the images in the dataset were resized to 750 × 500 pixels and kept in JPG file format. The annotations were performed by several team members using the tool computer vision annotation tool (CVAT). This tool allows a quite comfortable annotation of images that can later be used for training and testing different prediction models. When exporting the annotated images, they are accompanied with annotations stored in JavaScript object notation (JSON) file format. These annotations follow the common objects in context (COCO) format [28]. With this format, the coordinates of the annotations on the image and the object categories are stored for each image.

**Figure 3:**
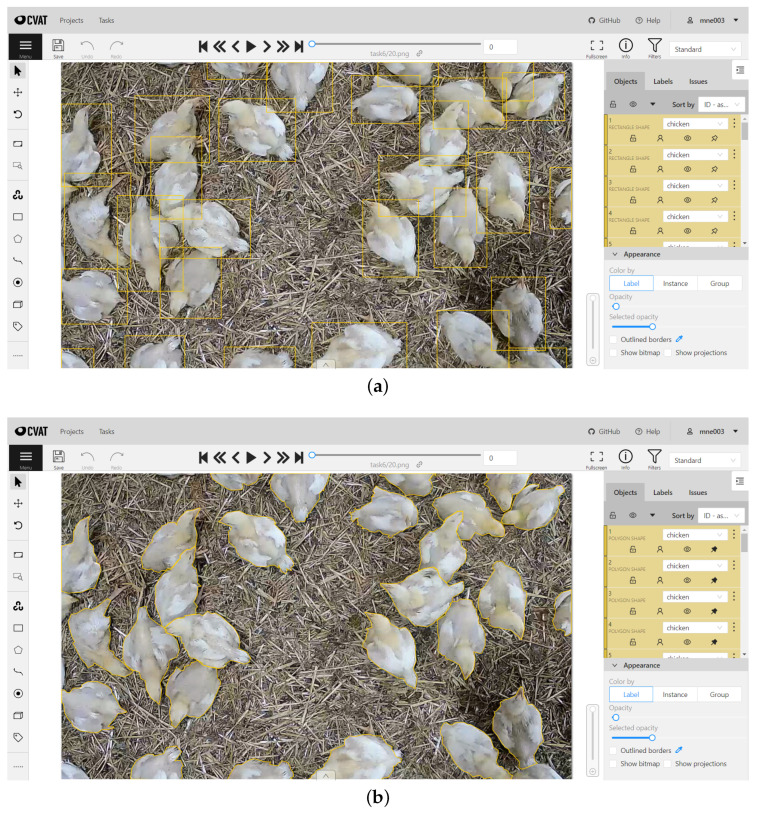
Annotation of the datasets using CVAT tool: (**a**) object detection labeling with rectangles (**b**) instance segmentation labeling using polygons.

For training the prediction models for detecting chickens in images, about 4000 images were annotated. This dataset was expanded using the RoboFlow augmentation tool from 4000 images to around 9000 annotated images. Of these 9000 images, 7550 were used for training, 725 for validation, and 725 for model testing. As for the task of training the models for instance segmentation, about 1000 images were annotated. This dataset was also augmented with the help of RoboFlow tool, which resulted in the total number of 1660 images. For the training, this dataset was split into 1300 images for training, 180 for validation, and 180 for model testing. The augmentation process for both datasets assumed extending datasets by relying on operations of rotation, brightness change (from −15% to +15%), saturation (from −10% to +10%), and crop (maximum 10%). We should note that the annotation process for instance segmentation is much more labor intensive as it requires drawing polygons over each instance of chicken as opposed to just drawing a rectangle for the dataset used for training detection models. The use of CVAT tool for the annotation is depicted in Figure 3.

### 3.2. Tools Selection and Setup

*Software Components.* The main software tools that are used for this research for model development is the Python programming language and Detectron2 package that includes various variants of deep neural networks [8]. Detectron2 package was developed by the company Facebook and it is based on the PyTorch package, which has an undisputed role in the implementation of neural networks. As mentioned in the related work, the selection of Detectron2 was encouraged by the research in [18,19,23], and the initial experimenting with Detectron2 gave good starting results with respect to the accuracy, required memory sizes, and prediction times. Detectron2 library provides Faster R-CNN network architectures for object detection and Mask R-CNN for instance segmentation [8,11]. Interesting discussions on comparison of Detectron2 and YOLO are given in [29,30]. Detectron2 is simple to install on Edge AI, in our case, the NVIDIA Jetson Nano. Detectron2 contains already trained models as shown in [31], but further training and improvement of prediction models is needed for real-life applications. The simulation scripts for the study were implemented in Python, the Pandas package is used for the tabular analysis of the results, and matplotlib was used for visualization. Initial computing was performed using Google’s Colab platform, but the main experimental part of the study was executed on HPC computing nodes.

*HPC Access.* HPC systems consist of clusters of computers that usually have better performance than the typical computers we use in everyday life. They provide better processing power, with a large number of cores, and typically have powerful RAM. Computers in these clusters (computing nodes) generally contain advanced graphics cards with graphical processing units (GPUs). This is important because GPUs have been shown to be more powerful than CPUs for training huge deep learning models with a large dataset [32]. The HPC system used for this research uses System Linux Utility for Resource Management (SLURM) whose main task is to manage HPC system resources [33]. With this system, there is usually one login node and several computing nodes. The system used for this research has two groups of computing nodes on which the experiments are performed. These nodes are CPU nodes and GPU nodes. There were 14 CPU nodes (node01 to node14), each of which was equipped with 2 Intel Xeon E5-2690v4 processors having 28 cores. Every node also has 512 gigabytes (GB) of fast DDR4 RAM. The GPU partition consists of 8 nodes (gpu01 to gpu08). In addition to everything a CPU node has, each GPU node in our system was equipped with 4 NVIDIA Tesla M60 GPU cards. A Tesla M60 card is made out of two physical NVIDIA Maxwell GPUs with a combined 16 GB of memory. Many applications perceive the card as two separate GPUs, appearing as total of 8 GPUs per node [34]. The training of the models in this study was executed on GPU nodes using different numbers of GPUs (1, 2, 4, or 8).

*Edge AI Platform.* Devices running predictive models belong to the class of edge AI internet of things (IoT) devices. More specifically, a Jetson Nano device with the following configuration was used: NVIDIA Maxwell GPU, Quad Core ARM Cortex-A57 processor, 4GB LPDDR4 memory, SD card 32 GB, and external USB with 64 GB. This device is connected to the Dahua Technology Smart H.265 IR Bullet network camera, which initially served to collect images in the field. Collected images were used to create the datasets and train the models. The configuration of the Jetson Nano device is quite limited in terms of hardware performance. Due to this fact, it is necessary to focus on the time duration of the prediction model, and not only on its precision. Another important parameter is the memory size of the model, but in our case there were no large oscillations in the size of the models, and the main parameters we focused on were the average precision and prediction times.

### 3.3. Experiment Execution

This study involved several computations to train the prediction models for object detection and instance segmentation using the datasets with images of chickens collected in poultry farms. The simulations focused on evaluating different deep neural network architectures based on faster R-CNN and mask R-CNN. Each network we tried was evaluated with different hyperparameter settings, batch sizes, and number of GPUs involved in computation. Around a total of 2000 network training computations were run for object detection and 2000 for instance segmentation. The key input parameters that were varied after selecting the number of graphics cards for training and the architecture of the neural network are: gamma, steps, max_iters, and ims_per_batch. The gamma and steps parameters are used to update the learning rate parameter starting from value base_learning_rate. Parameter max_iters represents the number of training epochs, while ims_per_batch represents a parameter known as batch size. Parameter steps indicate when the learning rate should be updated. In addition, parameter lr_policy was used for defining the update strategy of learning rate with value ”steps_with_decay” by the formula: $learing\text{\_}rate=base\text{\_}learning\text{\_}rate\times gamma^{step\text{\_}index}$ where step_index is a specific index in steps array. The rest of the settings were fixed for all experiments and their effects were not considered [4]. These runs were used to evaluate and select the specific architectures that would later be used to fine tune hyperparameters using AutoML.

The prediction models were evaluated by measuring average precision (AP), AP50, and AP75. Detectron2 uses the COCO evaluation metric [35] for AP which are averaged over multiple intersection over union (IoU) values, which itself is calculated by the formula: IoU=Area_of_overlap/Area_of_union [36]. In addition to the mentioned parameters, we also monitored the training time and prediction time. We monitored the training time parameter in order to compare the training duration of the deep learning model with different HPC system configurations. We used the PTime parameter to select the architecture of the neural network that should be used for model predictions. With the prediction time parameter, it was measured how much time it takes for 1 test image to be analyzed on the HPC system. Apart from the prediction time parameter, the AP parameter is also very important because this parameter indicates the overall model prediction quality. The best relationship between these two parameters led us to select the network architecture and then focus on its optimization.

The range in which the input parameters changed was selected by the brute-force principle. The gamma parameter takes values of 0.2, 0.5, 0.8, and 1. For the value of the gamma parameter 0, the learning rate has a fixed value. In rare situations, when gamma is 0, the best values of the AP parameter were obtained. The number of epochs varied from 500 to 1000. Various values for ims_per_batch were also tested to determine how this affects training time and prediction accuracy. For experiments on one GPU, the values of this parameter are taken as the powers of two (from the $2^{0}$ to $2^{8}$). For two GPUs, we used powers of two from $2^{1}$ to $2^{8}$, for four GPUs from $2^{2}$ to $2^{8}$, and finally, for eight graphics cards, from $2^{3}$ to $2^{8}$. It is important to emphasize that the parameter ims_per_batch cannot be less than the number of GPUs with which the experiment is carried out. In addition, the value of this parameter must be divisible by the number selected by the number of graphics for training models.

## 4. Results and Discussion

The simulation experiments were executed with several types of neural networks from Detectron2 based on faster R-CNN and mask R-CNN deep neural networks for object detection and instance segmentation, respectively. We then used the best value for the ratio between the accuracy AP and prediction time to select specific network architectures to be further refined using AutoML hyperparameter optimization.

### 4.1. Detection of Objects

For the detection of objects, faster R-CNN deep neural network architectures available in Detectron2 were used. Six different types of network architectures were evaluated for different values of input parameters while keeping values of the parameter gamma = 0.5 and the total number of steps = 1000. The results of this first simulation step are shown in Table 1. A large number of experiments were run for each architecture on a different number of GPUs, and the table shows only rows selected based on the highest AP achieved. In order to choose one network architecture, we considered both the AP and prediction time as we were focused on the prediction models that can be later ported to edge AI systems, i.e., NVIDIA Jetson Nano. This approach led to the selection of the faster_rcnn_R_101_FPN_3x network architecture, which offered the best initial ratio between the AP and prediction time.

Algorithm 1 provides step-by-step instructions in the form of pseudocode for creating the object detection prediction model using HPC and AI. The first part of the the algorithm deals with finding the neural network architecture with the best ratio between average precision (AP) and prediction time. Then, in the second part of the algorithm, for the selected neural network, a grid search is performed to determine the hyperparameters that are used to fine-tune the creation of the prediction model. At the end, the prediction model is saved for later use and porting on the edge AI platform.

**Algorithm 1** Object detection: implementation steps needed to select the network architecture and create the prediction model

 dataset←load(object_detection_dataset)



 optimal_batch←0



 selected_nn_arch←″″



 ratio←0



 gamma←0.5



 steps←1000



 base_lr←0.001


   **while**
num_gpus∈[1,2,4,8]
**do**
      **while** curr_nn_arch∈faster_r_cnn[r_101_c4_3x,r_101_fpn_3x,r_50_c4_1x,
   r_cnn_r_50_c4_3x,r_50_FPN_1x,x_101_32x8d_fpn_3x] **do**
            **while** $batch\text{\_}size\in [2^{0},2^{1},2^{2},2^{3},2^{4},2^{5},2^{6},2^{7},2^{8}]$ **do**
               train(num_gpus,curr_nn_arch,gamma,base_lr,batch_size,data,steps)
                **if** ratio < average_precision/prediction_time **then**
                   ratio←average_precision/prediction_time
                   optimal_batch_size←batch_size
                   selected_nn_arch←curr_nn_arch
               **end if**
             **end while**
           **end while**    
**end while** 

 detection_prediction_model←none



 best_average_precision←0


   **while**
num_gpus∈[1,2,4,8]
**do**
         **while** $batch\text{\_}size\in [2^{0},2^{1},2^{2},2^{3},2^{4},2^{5},2^{6},2^{7},2^{8}]$ **do**
                **while** gamma∈[0.2,0.5,0.8,1] **do**
              train(num_gpus,selected_nn_arch,gamma,base_lr,batch_size,data,steps)
               **if** best_average_precision < average_precision **then**
                    best_average_precision←average_precision
                    update(detection_prediction_model)
                            **end if**
                    **end while**
            **end while**
      **end while**

 save(detection_prediction_model)



Further refinement of the model was performed running computation on different configurations (number of GPUs) and by varying the parameters gamma (0.2, 0.5, 0.8, and 1) and batch size, all powers of 2 from 1 to 256 (Table 2). The number of steps was preset at 1000, since the AP did not significantly change for higher values. The accuracy achieved was AP = 84.62%, AP50 = 97.88%, and AP75 = 95.68%, which is comparable to the results reported in similar studies [13,16], and better than results reported in [20].

**Figure 4:**
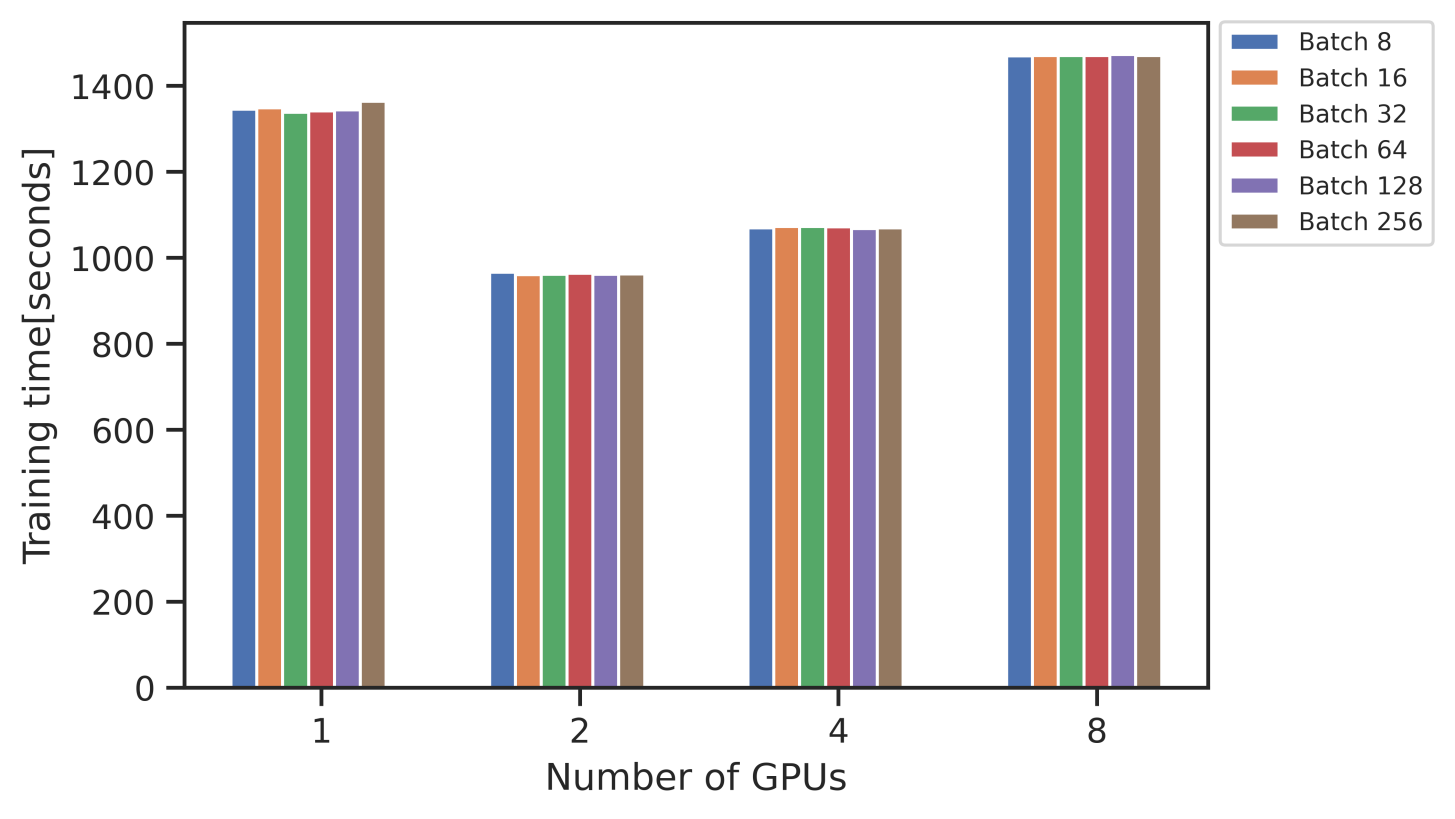
Training times for faster_rcnn_R_101_FPN_3x with 1000 epochs with different batch size.

The training times and potential benefits of using multiple GPUs can be observed in Figure 4. A faster training time was achieved with 2 and 4 GPUs, while a slower training time took place when using 8 GPUs, in comparison to 1 GPU. The explanation for this could be found in the communication between several GPUs, gradient synchronization, and the size of the dataset [37].

### 4.2. Instance Segmentation Results

For creating the instance segmentation prediction model, we ran computation using seven variants of mask R-CNN using different values of input parameters while keeping values of the parameter gamma = 0.5 and the total number of steps = 1000 (please see Algorithm 2). The results of this step are shown in Table 3. Similarly as for the detection, a large number of experiments was run for each architecture on different number of GPUs, and the table shows only rows selected based on the highest AP achieved. Again, we considered both the AP and prediction time as the aim was to find the prediction model that can be ported to NVIDIA Jetson Nano systems. This led to selection of the mask_rcnn_R_101_FPN_3x network architecture, which offered the best initial ratio between the AP and prediction time.

**Algorithm 2** Instance segmentation: implementation steps needed to select the network architecture and create the prediction model

 dataset←load(instance_segmentation_dataset)



 optimal_batch←0



 selected_nn_arch←″″



 ratio←0



 gamma←0.5



 steps←1000



 base_lr←0.001


   **while**
num_gpus∈[1,2,4,8]
**do**
      **while** curr_nn_arch∈mask_r_cnn[r_50_fpn_3x,r_101_c4_3x,r_101_fpn_3x,
   r_50_c4_1x,r_cnn_r_50_c4_3x,r_50_FPN_1x,x_101_32x8d_fpn_3x] **do**
            **while** $batch\text{\_}size\in [2^{0},2^{1},2^{2},2^{3},2^{4},2^{5},2^{6},2^{7},2^{8}]$ **do**
                 train(num_gpus,curr_nn_arch,gamma,base_lr,batch_size,data,steps)
                **if** ratio < average_precision/prediction_time **then**
                   ratio←average_precision/prediction_time
                   optimal_batch_size←batch_size
                   selected_nn_arch←curr_nn_arch
               **end if**
             **end while**
           **end while**    
**end while** 

 segmentation_prediction_model←none



 best_average_precision←0


   **while**
num_gpus∈[1,2,4,8]
**do**
         **while** $batch\text{\_}size\in [2^{0},2^{1},2^{2},2^{3},2^{4},2^{5},2^{6},2^{7},2^{8}]$ **do**
                **while** gamma∈[0.2,0.5,0.8,1] **do**
              train(num_gpus,selected_nn_arch,gamma,base_lr,batch_size,data,steps)
               **if** best_average_precision < average_precision **then**
                    best_average_precision←average_precision
                    update(segmentation_prediction_model)
                            **end if**
                    **end while**
            **end while**
      **end while**

 save(segmentation_prediction_model)



Again, using the same approach as for detection, AutoML refinement of the model was performed running computation on different configurations (number of GPUs) and by varying the parameters gamma (0.2, 0.5, 0.8, and 1) and batch size, all powers of 2 from 1 to 256, results shown in Table 4. The number of steps was fixed at 1000. The final model achieved accuracy of AP = 89.73%, AP50 = 98.35%, and AP75 = 96.23%. We have not identified a research to directly compare, but the results are comparable with a similar research on instance segmentation in geese images [26]. The authors were developing their own model and compared it to different instance segmentation networks and in this process reporter values around 81%, 96%, 90% for AP, AP50 and AP75, respectively. For their mode QueryPNet model they reported AP50 = 96.3%.

**Figure 5:**
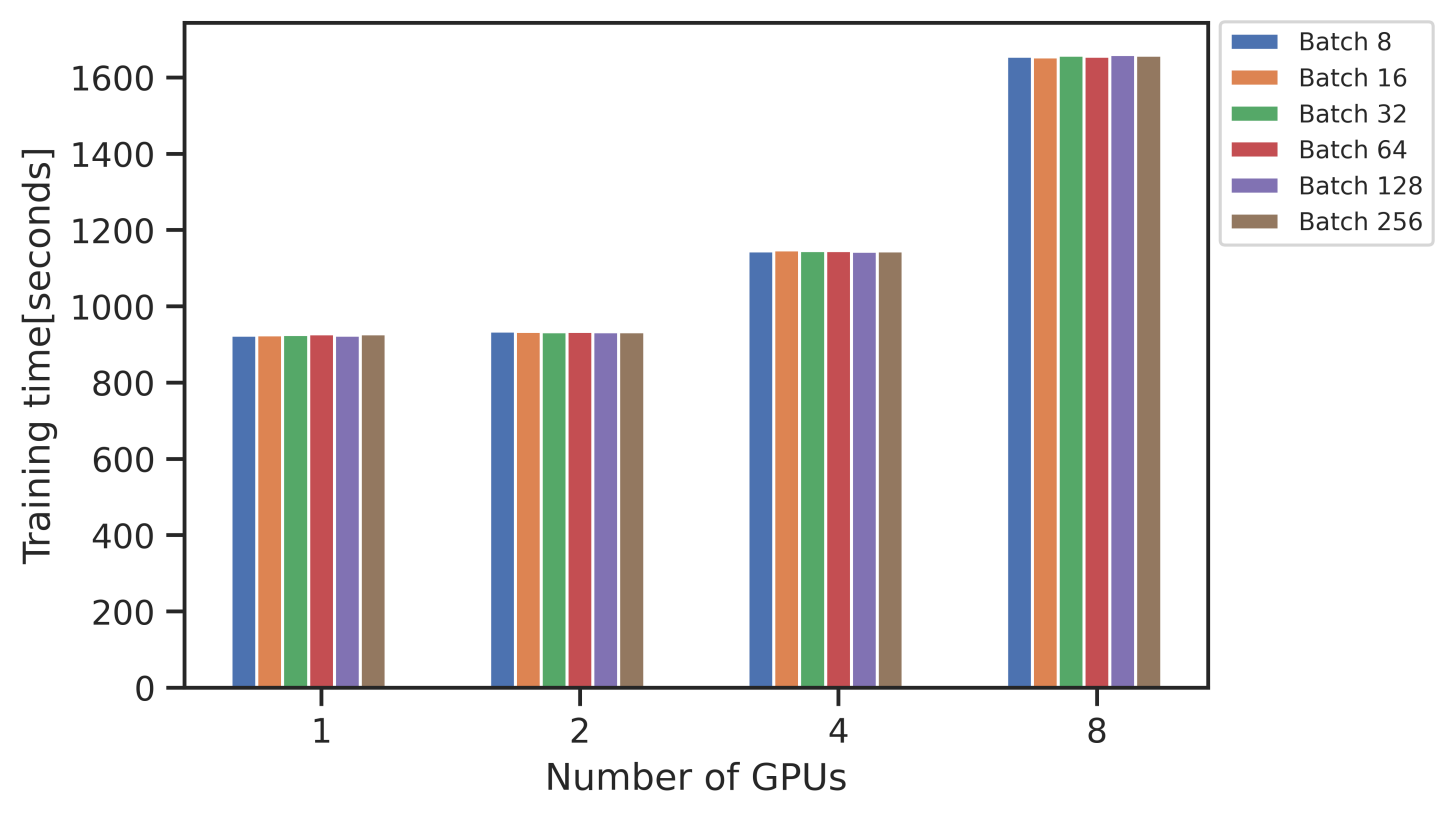
Training times for mask_rcnn_R_101_FPN_3x with 1000 epochs with different batch size.

Figure 5 shows that running the computations on multiple GPUs in this case did not result in faster training times. This was not unexpected because the training dataset contained less annotated data available to train the model [37].

### 4.3. Initial Field Evaluation and Validation

**Figure 6:**
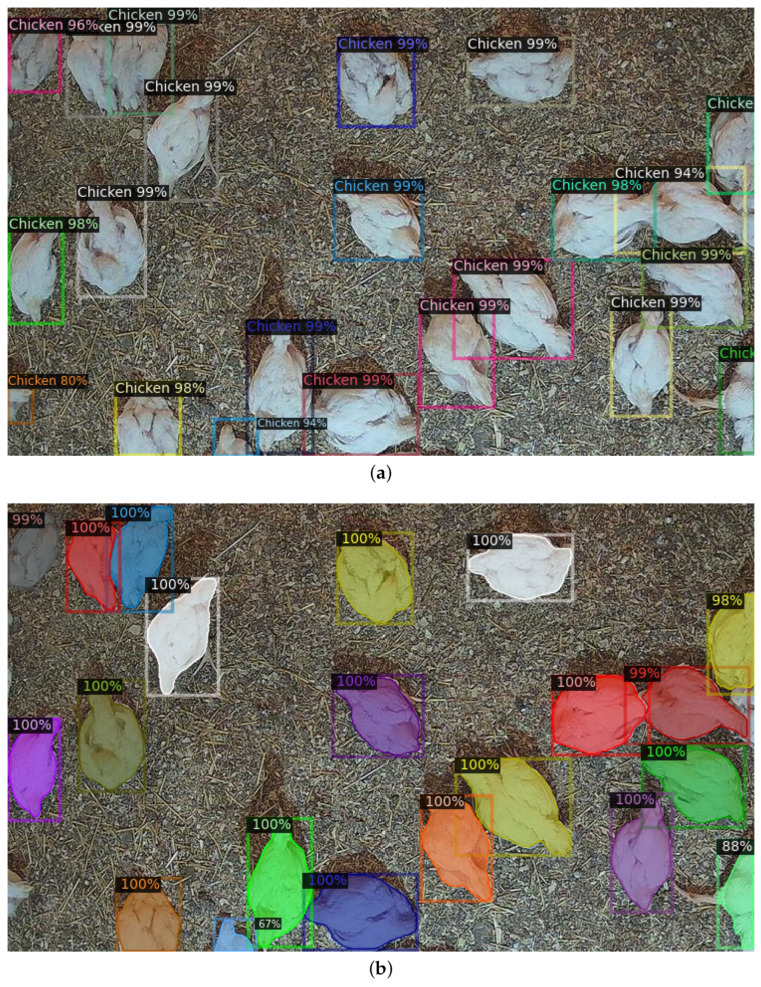
Field verification and validation using the models on the edge AI device at the end user site: (**a**) object detection (**b**) instance segmentation.

It is worth mentioning that the creation and validation of the prediction models was not limited to simulation experiments only. The prediction models were successfully ported onto the edge AI devices, in this case NVIDIA Jetson Nano systems equipped with camera sensors. The camera would take video clips every 15 min. From each video clip, one or more image frames could be extracted depending on the desired settings. Finally, execution of prediction models for each frame would take up to 15 s, which is satisfactory for real-life applications in IoT systems. The field verification and validation of prediction models is illustrated in Figure 6.

**Figure 7:**
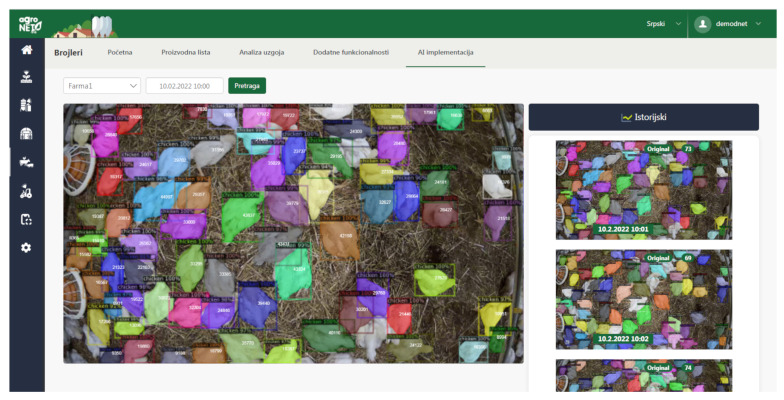
Integration with an existing digital farming platform.

The resulting images and extracted information can then be sent to the IoT platform [38]. During this experimental study, we successfully connected the edge AI nodes with the actual digital farming solution as illustrated in Figure 7. In this case, for the experiment, we were sending both the images and extracted knowledge using the prediction models. However, the future uses will be configured to only send the extracted results in order to reduce the data throughput. Otherwise, the use of edge AI would not be fully justified as the prediction models could reside in the cloud with the platform.

In addition, it is important to note that the information coming from these sensor nodes with cameras and AI models should be seen as an input data for new and improved decision support modules, developed within the digital poultry farm management system, that may be focusing on determining the number of chickens, detecting of dead chickens, assessing their weight, or detecting issues in uneven growth. Most of these functions aim for early detection of health issues and prevention of disease spreading. However, these decision support functions are beyond the scope of this paper.

## 5. Conclusions

The paper provides a real-life use case for AI/ML, HPC, and edge AI in smart agriculture, namely to develop computer vision software modules that can be integrated into smart agriculture solutions for poultry farms. The research study focused on the models for object detection and object instance segmentation, where the objects are chickens in an image taken in poultry farms using edge devices equipped with camera sensors. Detailed step-by-step instructions in the form of pseudocode are provided to explain how to implement the process of computing the prediction models. These models are envisioned to become a part of a sensor edge AI kit that can be connected with smart IoT based farming platforms in order to improve the health assessment of chickens, disease detection, and prevention of disease spreading. Further research will be performed to not only expand the datasets and improve the accuracy of the models by testing different network architectures and libraries, but also to explore creation of new decision support tools based on the output from the models. In addition, the future work will include exploring model pruning techniques to optimize the models, especially for the edge side of the solution.

The main contributions are summarized below:

- Conceptual approach and methodology how to combine offline computing using HPC and AI to develop and refine prediction models, and online processing of the data using these models ported onto the edge AI devices. This includes the preparation of the data set and selection of software tools that enabled creation of the models that have necessary accuracy with a possibility to be ported and used on the edge AI IoT nodes, and integrated with an existing IoT based digital platform for poultry farms. The experimenting is performed using Python and Detectron 2 library.
- For object detection, the Faster R-CNN deep convolutional network was used. HPC was used to compute and test six different architectures offered by Detectron2. After testing with different parameters, the faster_rccn_R_101_FPN_3x network architecture was selected as one offering a good balance between accuracy and prediction time. Further experimenting with the hyperparameter optimization resulted in a prediction model with AP = 84.62%, AP50 = 97.88%, and AP75 = 95.68%.
- The mask R-CNN network was selected for the instance segmentation problem. HPC was used to test seven different architectures and mask_rcnn_R_101_FPN_3x was selected based on the accuracy and prediction time. With further refining of the hyperparameters, we obtained a model with AP = 89.73%, AP50 = 98.35%, and AP75 = 96.23%.
- For the initial field evaluation, these prediction models were ported onto NVIDIA Jetson Nano devices equipped with cameras. These sensor prototypes were integrated with an actual IoT based platform installed in a real-life farm. The setup is configured so that it can be also used to collect new images to enhance the dataset for future research.

## Figures and Tables

**Table 1:** Object detection: evaluation of different Faster R-CNN network architectures in Detectron2, gamma = 0.5, steps = 1000.

Number of GPUs	Neural Network Arhitecture	Training Time [min]	Batch Size	Validation Loss	AP	AP50	AP75	Prediction Time [s]
1	faster_r_cnn_R_101_C4_3x	50:50	128	0.27	83.92	97.79	94.31	0.6
	**faster_r_cnn_R_101_FPN_3x**	**22:44**	**256**	**0.31**	**82.96**	**97.8**	**94.3**	**0.2**
	faster_r_cnn_R_50_C4_1x	44:44	64	0.34	81.74	97.68	93.1	0.6
	faster_r_cnn_R_50_C4_3x	44:41	32	0.31	82.78	97.76	94.32	0.6
	faster_r_cnn_R_50_FPN_1x	16:43	8	0.36	80.75	97.7	93.2	0.2
	faster_r_cnn_X_101_32x8d_FPN_3x	42:05	8	0.31	83.39	97.81	94.42	0.4
2	faster_r_cnn_R_101_C4_3x	34:16	2	0.27	84.1	97.81	94.43	0.6
	faster_r_cnn_R_101_FPN_3x	15:58	4	0.3	82.9	97.82	94.4	0.2
	faster_r_cnn_R_50_C4_1x	30:03	4	0.34	82.22	97.73	93.9	0.6
	faster_r_cnn_R_50_C4_3x	30:0	256	0.31	82.73	97.78	94.2	0.6
	faster_r_cnn_R_50_FPN_1x	12:10	4	0.35	81.18	97.74	93.22	0.2
	faster_r_cnn_X_101_32x8d_FPN_3x	31:00	16	0.3	83.34	97.86	94.51	0.4
4	faster_r_cnn_R_101_C4_3x	32:19	256	0.26	84.1	97.8	94.3	0.6
	faster_r_cnn_R_101_FPN_3x	17:56	4	0.29	83.14	97.81	94.44	0.2
	faster_r_cnn_R_50_C4_1x	28:36	256	0.33	82.43	97.72	93.13	0.6
	faster_r_cnn_R_50_C4_3x	28:30	256	0.3	82.58	97.8	94.3	0.6
	faster_r_cnn_R_50_FPN_1x	14:25	128	0.33	81.34	97.74	93.22	0.2
	faster_r_cnn_X_101_32x8d_FPN_3x	31:17	64	0.31	83.47	97.82	94.56	0.4
8	faster_r_cnn_R_101_C4_3x	36:10	256	0.25	83.8	97.81	94.3	0.6
	faster_r_cnn_R_101_FPN_3x	13:57	16	0.28	83.24	97.81	94.43	0.2
	faster_r_cnn_R_50_C4_1x	32:28	256	0.32	82.43	97.72	93.21	0.6
	faster_r_cnn_R_50_C4_3x	32:31	256	0.29	82.83	97.8	94.3	0.6
	faster_r_cnn_R_50_FPN_1x	20:57	8	0.32	81.61	97.76	94.15	0.2
	faster_r_cnn_X_101_32x8d_FPN_3x	37:36	32	0.32	83.59	97.85	94.56	0.4

**Table 2:** Object detection: hyperparameter optimization results for faster_rcnn_R_101_FPN_3x, 1000 epochs, for different values of gamma and batch sizes.

Number of GPUs	Training Time [min]	Gamma	Batch Size	Validation Loss	AP	AP50	AP75
1	22:20	0.8	1	0.31	83.7	97.86	94.62
2	15:45	0.8	128	0.3	84.11	97.85	95.43
4	17:53	1	256	0.27	84.56	97.88	95.59
8	24:25	1	8	0.26	84.62	97.88	95.68

**Table 3:** Instance segmentation evaluation of different Mask R-CNN network architectures in Detectron2, gamma = 0.5 and steps = 1000.

Number of GPUs	Neural Network Arhitecture	Training Time [min]	Batch Size	Validation Loss	AP	AP50	AP75	Prediction Time [s]
1	mask_rcnn_R_50_FPN_3x	11:50	8	0.48	87.5	97.45	95.19	0.2
	mask_rcnn_R_101_C4_3x	31:39	32	0.37	85.7	98.29	95.93	0.7
	**mask_rcnn_R_101_FPN_3x**	**15:24**	**8**	**0.43**	**88.3**	**98.3**	**96.15**	**0.3**
	mask_rcnn_R_50_C4_1x	28:10	128	0.46	84.4	97.21	94.96	0.6
	mask_rcnn_R_50_C4_3x	28:11	0.8	0.41	84.8	97.26	95.13	0.6
	mask_rcnn_R_50_FPN_1x	11:58	8	0.51	86.7	97.33	95.01	0.2
	mask_rcnn_X_101_32x8d_FPN_3x	28:47	64	0.42	88.5	98.28	96.50	0.6
2	mask_rcnn_R_50_FPN_3x	12:21	2	0.47	87.4	97.44	95.99	0.2
	mask_rcnn_R_101_C4_3x	28:47	128	0.37	85.69	98.31	96.07	0.7
	mask_rcnn_R_101_FPN_3x	15:35	8	0.43	88.57	98.34	96.19	0.3
	mask_rcnn_R_50_C4_1x	25:31	256	0.46	84.48	97.21	94.96	0.6
	mask_rcnn_R_50_C4_3x	25:27	128	0.43	85.00	97.28	95.12	0.6
	mask_rcnn_R_50_FPN_1x	12:23	8	0.5	86.94	97.32	95.05	0.2
	mask_rcnn_X_101_32x8d_FPN_3x	27:55	2	0.42	88.33	97.43	96.1	0.7
4	mask_rcnn_R_50_FPN_3x	15:51	8	0.41	87.6	98.23	96.02	0.2
	mask_rcnn_R_101_C4_3x	31:43	16	0.34	85.8	98.26	96.08	0.7
	mask_rcnn_R_101_FPN_3x	19.06	64	0.38	88.67	98.36	96.19	0.3
	mask_rcnn_R_50_C4_1x	28:07	32	0.4	84.51	97.22	94.97	0.6
	mask_rcnn_R_50_C4_3x	28:11	4	0.38	84.8	97.31	95.07	0.6
	mask_rcnn_R_50_FPN_1x	15:51	32	0.44	86.7	97.35	95.01	0.2
	mask_rcnn_X_101_32x8d_FPN_3x	31:59	4	0.38	88.3	97.38	96.05	0.7
8	mask_rcnn_R_50_FPN_3x	24:22	64	0.41	87.67	98.24	96.08	0.2
	mask_rcnn_R_101_C4_3x	37:58	64	0.35	85.66	98.27	96.07	0.7
	mask_rcnn_R_101_FPN_3x	27:38	32	0.41	88.29	97.5	96.11	0.3
	mask_rcnn_R_50_C4_1x	34:14	16	0.42	84.47	97.23	94.89	0.7
	mask_rcnn_R_50_C4_3x	34:16	8	0.38	84.88	97.29	95.06	0.7
	mask_rcnn_R_50_FPN_1x	24:23	128	0.44	86.74	97.37	95.08	0.2
	mask_rcnn_X_101_32x8d_FPN_3x	40:14	8	0.37	88.45	97.46	96.13	0.7

**Table 4:** Instance segmentation, hyperparameter optimization results for mask_rcnn_R_101_FPN_3x, 1000 epochs, for different values of gamma and batch sizes.

Number of GPUs	Training Time [min]	Gamma	Batch Size	Validation Loss	AP	AP50	AP75
1	15:17	0.8	2	0.44	89.13	97.41	94.09
2	15:38	1	4	0.43	89.3	98.3	96.16
4	19:09	1	4	0.36	89.73	98.35	96.23
8	27:37	1	256	0.35	89.49	97.47	96.16

## Data Availability

Not applicable.

## Abbreviations

The following abbreviations are used in this manuscript:

AI	Artificial Intelligence
AP	Average Precision
AutoML	Automated Machine Learning
CNN	Convolutional Neural Network
CVAT	Computer Vision Annotation Tool
COCO	Common Object in Context
CPU	Central Processing Unit
HPC	High-Performance Computing
HPO	Hyperparameter Optimization
GPU	Graphics Processing Unit
IoT	Internet of Things
JSON	JavaScript Object Notation
ML	Machine Learning
RAM	Random Access Memory
SSD	Single Shoot Detector
USB	Universal Serial Bus
YOLO	You Only Look Once
